# Integrated bioinformatics analysis of noncoding RNAs with tumor immune microenvironment in gastric cancer

**DOI:** 10.1038/s41598-023-41444-3

**Published:** 2023-09-11

**Authors:** Jun Xu, Shengnan Hu, Qiuli Chen, Lilu Shu, Peter Wang, Jianjiang Wang

**Affiliations:** 1 First People’s Hospital of Hangzhou Lin’an District, Affiliated Lin’an People’s Hospital, Hangzhou Medical College, Hangzhou, China; 2 Department of Research and Development, Zhejiang Zhongwei Medical Research Center, Hangzhou, 310018 Zhejiang China

**Keywords:** Cancer, Cancer microenvironment, Gastrointestinal cancer

## Abstract

In recent years, molecular and genetic research hotspots of gastric cancer have been investigated, including microRNAs, long noncoding RNAs (lncRNAs) and messenger RNA (mRNAs). The study on the role of lncRNAs may help to develop personalized treatment and identify potential prognostic biomarkers in gastric cancer. The RNA-seq and miRNA-seq data of gastric cancer were downloaded from the TCGA database. Differential analysis of RNA expression between gastric cancer samples and normal samples was performed using the edgeR package. The ceRNA regulatory network was visualized using Cytoscape. KEGG pathway analysis of mRNAs in the ceRNA network was performed using the clusterProfiler package. CIBERSORT was used to distinguish 22 immune cell types and the prognosis-related genes and immune cells were determined using Kaplan-Meier and Cox proportional hazard analyses. To estimate these nomograms, we used receiver operating characteristic and calibration curve studies. The ceRNA regulation network of gastric cancer was built in this study, and the genes in the network were analyzed for prognosis. A total of 980 lncRNAs were differentially expressed, of which 774 were upregulated and 206 were downregulated. A survival study identified 15 genes associated with gastric cancer prognosis, including VCAN-AS1, SERPINE1, AL139002.1, LINC00326, AC018781.1, C15orf54, hsa-miR-145. Monocytes and Neutrophils were associated with the survival rate of gastric cancer. Our research uncovers new ceRNA network for the detection, treatment, and monitoring of gastric cancer.

## Introduction

Gastric cancer is one of the most common gastrointestinal malignancies in the world^1^. There were an estimated 26,380 new cases and 11,090 deaths of gastric cancer in the United States in 2022^2^. The incidence of gastric cancer varies significantly by region: highest in East Asia, Eastern Europe and South America, lowest in North and South Africa^3^. Helicobacter Pylori (HP) infection is the most important risk factor for gastric tumorigenesis^4^. Approximately 2 billion people worldwide are infected with HP, of which about 1 million will develop gastric cancer^5^. Hence, HP infection significantly increases the incidence of gastric cancer^6^. In addition to HP infection, gender, heredity, severe lack of sleep, irregular diet, excessive work and psychological stress can all contribute to the increased incidence of gastric cancer^7^.

Gastric cancer has low early diagnosis rate and 5 years survival rate^8^. Advanced gastric cancer can be metastasized to liver, pancreas, omentum, esophagus, bile duct and lymph node, and the treatment effect is poor^9^. Therefore, it is very important to find effective early diagnosis biomarkers and therapeutic targets for gastric cancer. Studies have found that the formation of gastric cancer is a complex process involving multiple factors and is usually associated with abnormal gene expressions^10^. At the same time, some studies have proved that noncoding RNAs (ncRNAs) are related to the development of cancer, and these studies have been continuously reported in various cancers^11,12^.

Long non-coding RNAs (lncRNAs) belong to noncoding RNAs with a length of more than 200 nucleotides^13–15^. Recently, some researchers found that many lncRNAs are new regulators of gene expression, and aberrant expression of some lncRNAs is involved in the pathogenesis and progression of malignant tumors^16–18^. These new lncRNAs may be developed into new clinical biomarkers or new therapeutic targets^19–21^. A large number of lncRNAs are associated with various cancers and have antitumor or oncogenic functions^22–24^. Altered lncRNA expression and its mutations are associated with tumorigenesis and metastasis^25^. Studies have confirmed that both lncRNAs and mRNAs can bind to miRNAs and regulate each other.

LncRNAs have been implicated in facilitating the evasion of immune surveillance by tumor cells through multiple mechanisms, including the promotion of an immunosuppressive microenvironment^26–28^. Multiple studies used comprehensive analysis to determine the functions of various lncRNAs in tumor immune microenvironment (TIME) in various cancers, such as colon cancer^29^, clear cell renal cell carcinoma^30^, breast cancer^31,32^, lung cancer^33,34^, thyroid cancer^35^, adrenocortical adenocarcinoma^36^, bladder cancer^37^, and head and neck squamous cell carcinoma^38^. LncRNA NKILA has demonstrated the ability to initiate apoptosis in tumor-specific T cells, thereby impeding their capacity to infiltrate the tumor, leading to tumor immune evasion^39^. LncRNA FENDRR mediated TIME and tumor suppression in non-small cell lung cancer (NSCLC)^40^. LncRNA RP5-881L22.5 was reported to play a crucial role in the TIME of tumors and in the colorectal cancer progression^41^. LncRNA WDFY3-AS2 has been identified to correlate with an immunosuppressive phenotype in the TIME in oral squamous cell carcinoma (OSCC)^42^. WDFY3-AS2 regulated Wnt/β-catenin pathway and promoted proliferation and metastasis in OSCC^42^. However, the role of lncRNAs in TIME of gastric cancer has not been elucidated. Therefore, to further investigate the molecular mechanisms underlying the development of gastric cancer, our study investigated the process of lncRNAs binding to miRNAs, mRNAs and proteins in gastric cancer to regulate gene expression. Moreover, we tried to comprehensively evaluate the correlation between lncRNAs and the prognosis and immune cell infiltration level of gastric cancer patients.

## Results

### Differentially expressed RNAs

**Figure 1:**
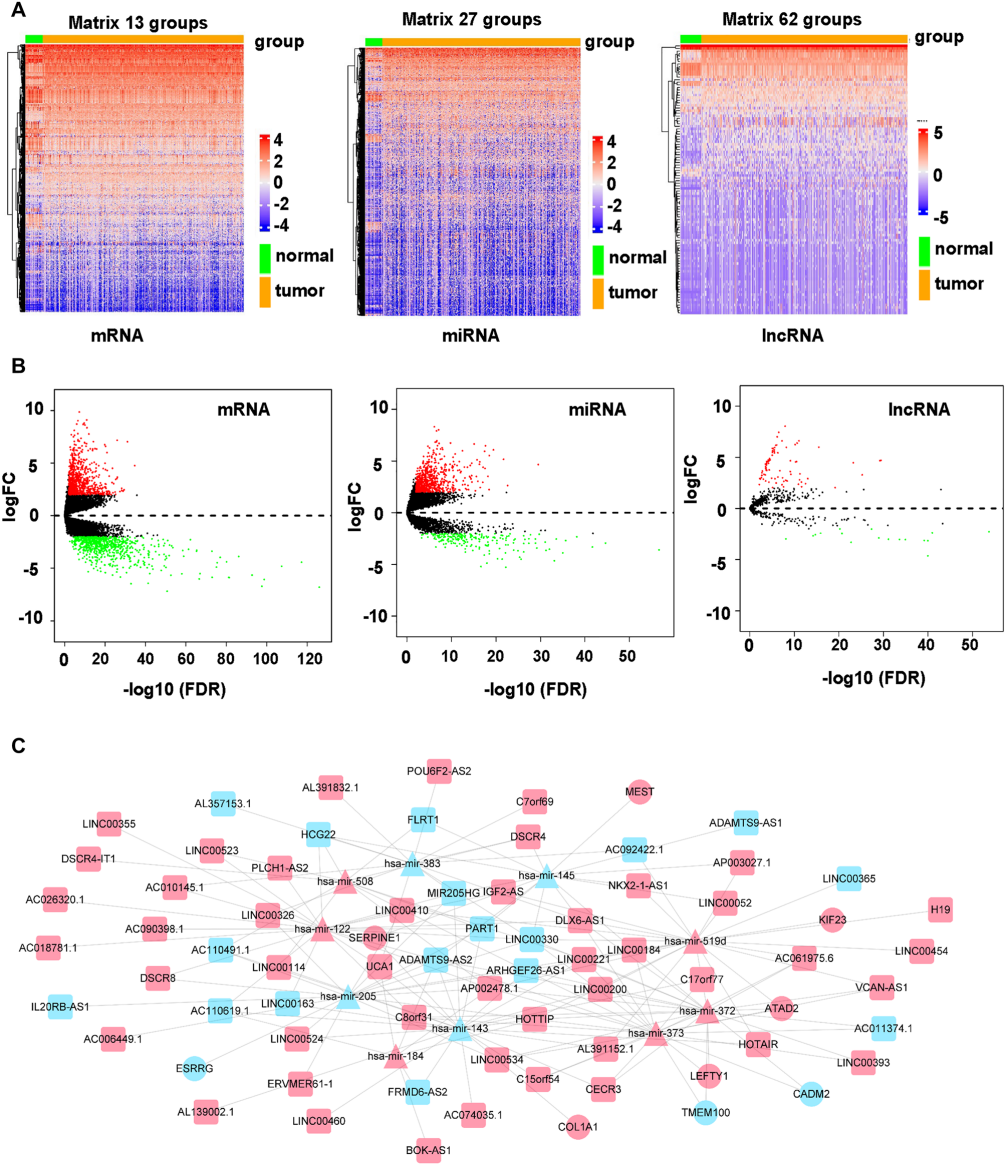
Volcano plots and heatmaps of differentially expressed mRNAs, miRNAs, and lncRNAs. (**A**) Volcano plot of differentially expressed mRNAs, miRNAs and lncRNAs. (**B**) Heatmap of differentially expressed mRNAs, miRNAs and lncRNAs. (**C**) LncRNA-miRNA-mRNA ceRNA network generated by Cytoscape. Red: upregulated RNAs. Green: downregulated RNAs. Triangle: miRNA. Diamond: lncRNA. Circular: mRNA.

In this study, 980 differentially expressed lncRNAs, 104 differentially expressed miRNAs, and 1639 differentially expressed mRNAs were obtained by “DESeq2” analysis with cutoff (|logFC|≥ 2 and *p* < 0.01). We found that among 980 DElncRNAs, 774 lncRNAs were upregulated, while 206 lncRNAs were downregulated. In addition, 87 miRNAs were elevated and 17 miRNAs were reduced. 870 mRNAs were upregulated, whereas 769 mRNAs were downregulated. Heatmaps of mRNAs, miRNAs, and lncRNAs were shown in Fig. 1A. The volcano plot of mRNAs, miRNAs, and lncRNAs was shown in Fig. 1B.

### ceRNA regulatory network

**Figure 2:**
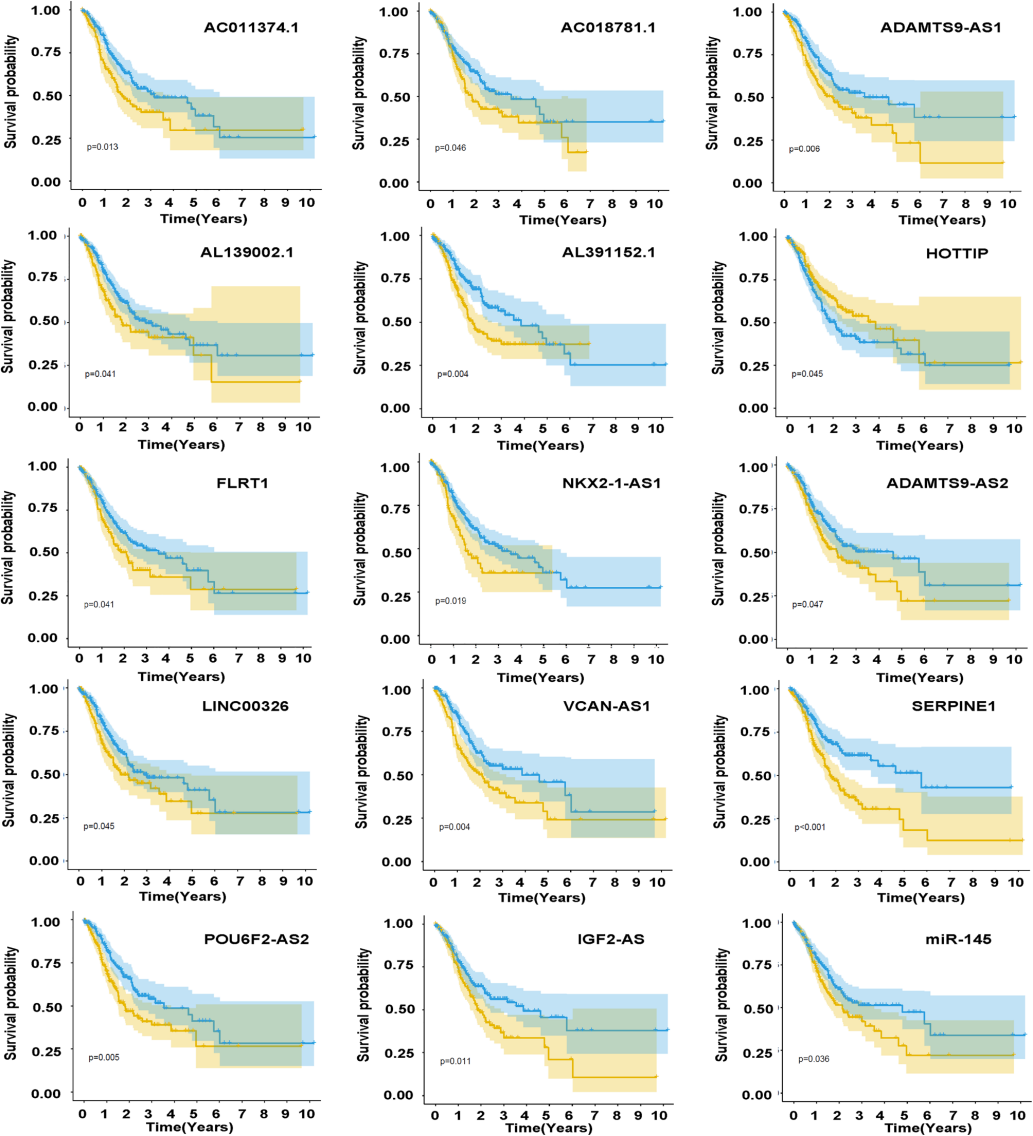
Prognosis-related genes were illustrated by the K-M survival evaluation.

To further understand the role of RNAs in gastric cancer, the interactions between miRNA-mRNA and lncRNA-miRNA served as the foundation for the development of the ceRNA network. The Cytoscape visualization of the ceRNA regulatory network was shown (Fig. 1C). The association between the prognosis and biomarkers in the STAD-linked ceRNA network was examined by the K-M survival evaluation. These analyses showed 15 genes were implicated in the prognosis of gastric cancer, including AC011374.1, AC018781.1, ADAMTS9-AS1, AL139002.1, AL391152.1, HOTTIP, FLRT1, NKX2-1-AS1, ADAMTS9-AS2, LINC00326, VCAN-AS1, SERPINE1, POU6F2-AS2, IGF2-AS, and miR-145 (Fig. 2).

### Prognosis of related genes

**Figure 3:**
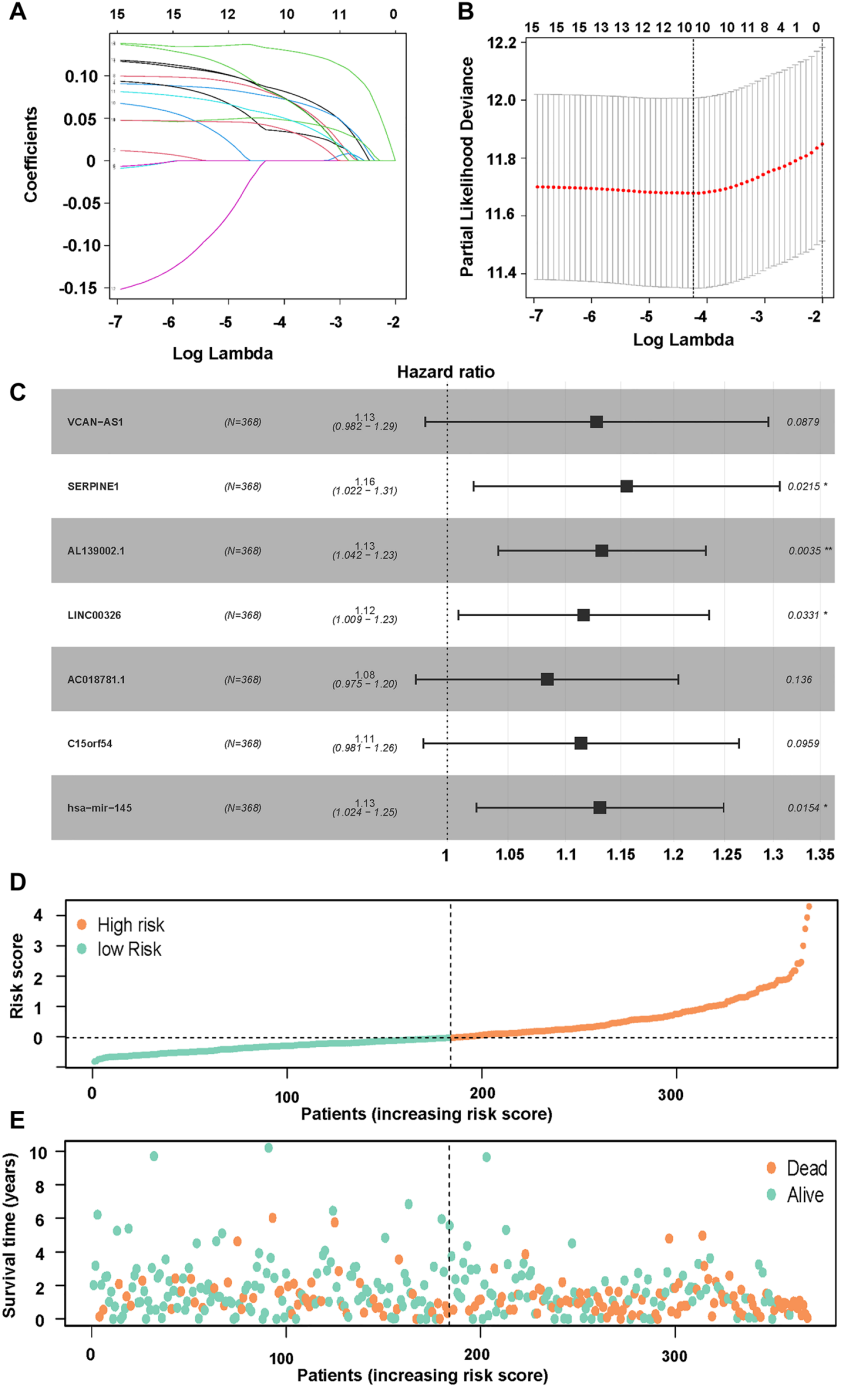
LASSO and multivariate regression models screening genes. (**A**) LASSO coefficient profiles of genes in STAD; dotted vertical lines were drawn at the optimal values by using the minimum criteria. (**B**) LASSO coefficient profiles of the candidate OS-related GRGs with nonzero coefficients determined by the optimal lambda. A coefficient profile plot was produced against the logλ sequence. (**C**) Forest plot of several genes was involved in the ceRNA network. (**D**) Risk score analyses of GC patients in the training cohort based on the seven-GRGs signature. Distribution of risk scores per patient. (**E**) Relationships between survival status and survival times of GC patients ranked by risk score. The black dotted line represents the optimum cut-off point dividing patients into low and high-risk groups.

Lasso regression analysis was performed, and two plots were generated. The variation of coefficients at different log Lambda values was depicted (Fig. 3A). The horizontal axis represented log Lambda, while the vertical axis represented the values of the coefficients. The changes in partial likelihood deviance at different log Lambda values were showcased (Fig. 3B). The horizontal axis represented log Lambda, and the vertical axis represented the values of the partial likelihood deviance. VCAN-AS1, SERPINE1, AL139002.1, LINC00326, AC018781.1, C15orf54, hsa-miR-145 were obtained by Multivariate Cox regression analysis (Fig. 3C). The scores were then calculated, and the patients were classified into high-risk and low-risk groups based on the median risk score in the training set (Fig. 3D). The relationships between survival status and survival times of gastric cancer patients, ordered by their respective risk scores, were displayed (Fig. 3E).

**Figure 4:**
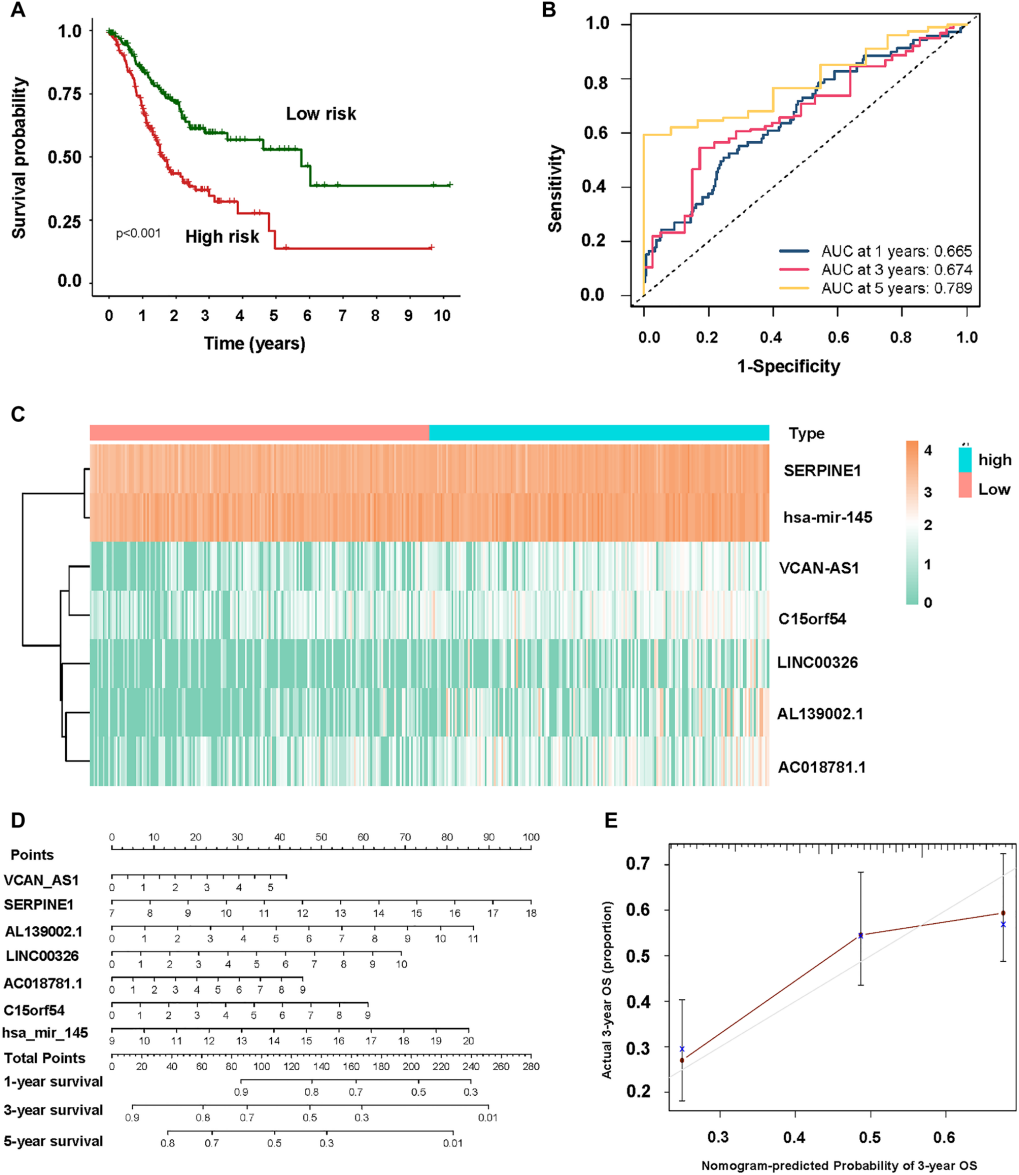
The association of ceRNAs and survival. (**A**) KM analysis of the high-risk and low-risk groups. (**B**) Time-dependent receiver operating characteristic (ROC) analysis for OS prediction of prediction model. (**C**) Heatmap of seven differentially expressed genes. (**D**) Nomogram based on multiple Cox regression. (**E**) Calibration curve for 3 years survival.

The high -risk group had a worse chance of survival than the low-risk group (Fig. 4A). The AUC at 1 year, 3 years, 5 years were 0.665, 0.674 and 0.789 (Fig. 4B). The gene SERPINE1 was highly expressed in both high and low-risk groups (Fig. 4C). The Nomogram was established based on the independent prognostic indicators, which predicts survival rates for the first, 3 to 5 years. The genes in this nomogram model were VCAN-AS1, SERPINE1, AL139002.1, LINC00326, AC018781.1, C15orf54, hsa-miR-145 (Figs. 4D). Moreover, the calibration curve for nomogram models showed a good agreement between the actual survival rate and the predicted 3 years overall survival rate, suggesting that this model was accurate in its predictions (Fig. 4E).

### Investigation of immune infiltration and gastric cancer

**Figure 5:**
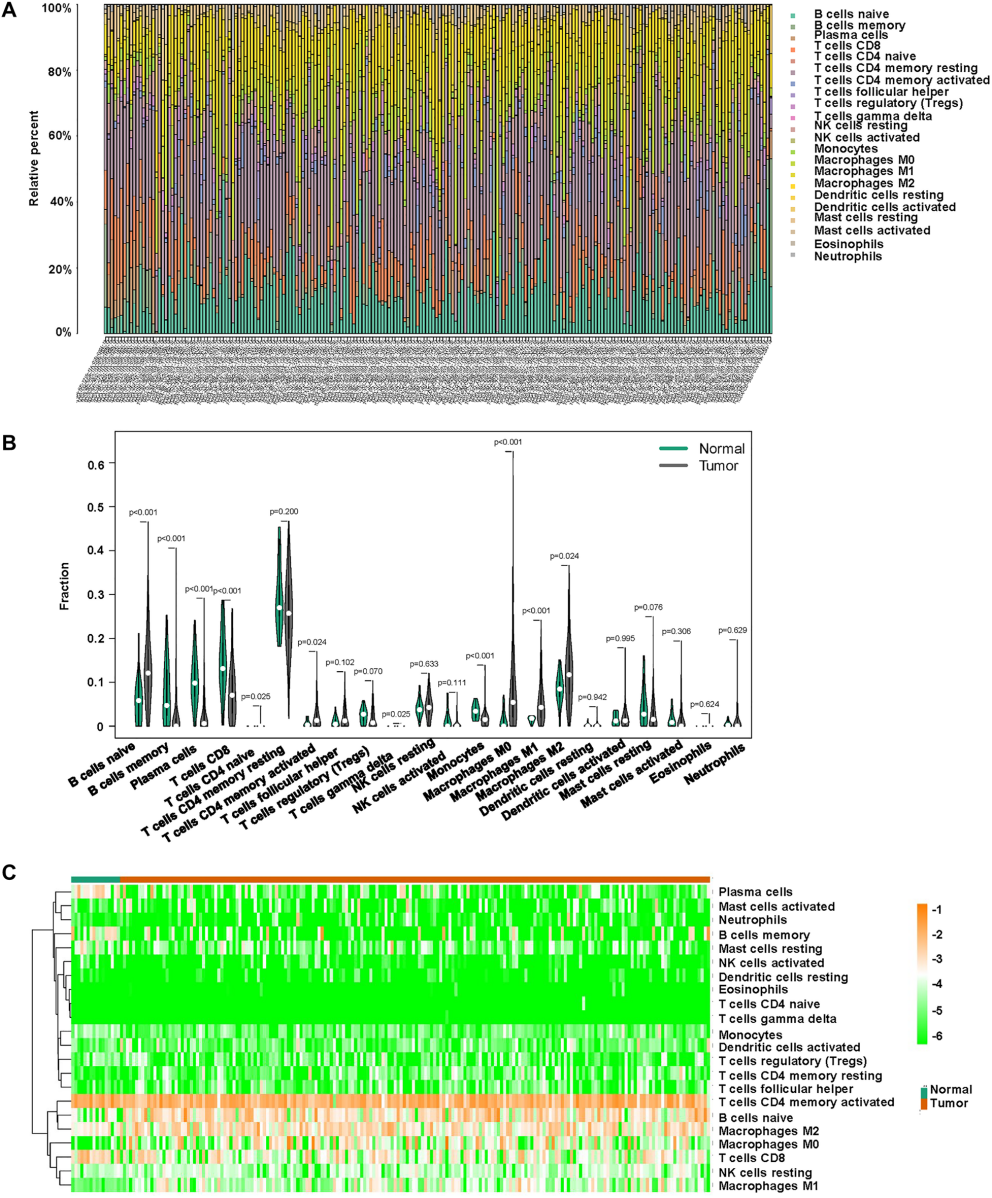
The association of immune cells and STAD. (**A**) The percentage of 22 immune cell subpopulations in STAD patients. (**B**) The fraction of immune cells in STAD patients. (**C**) Heatmap of immune infiltration between normal and tumor groups.

**Figure 6:**
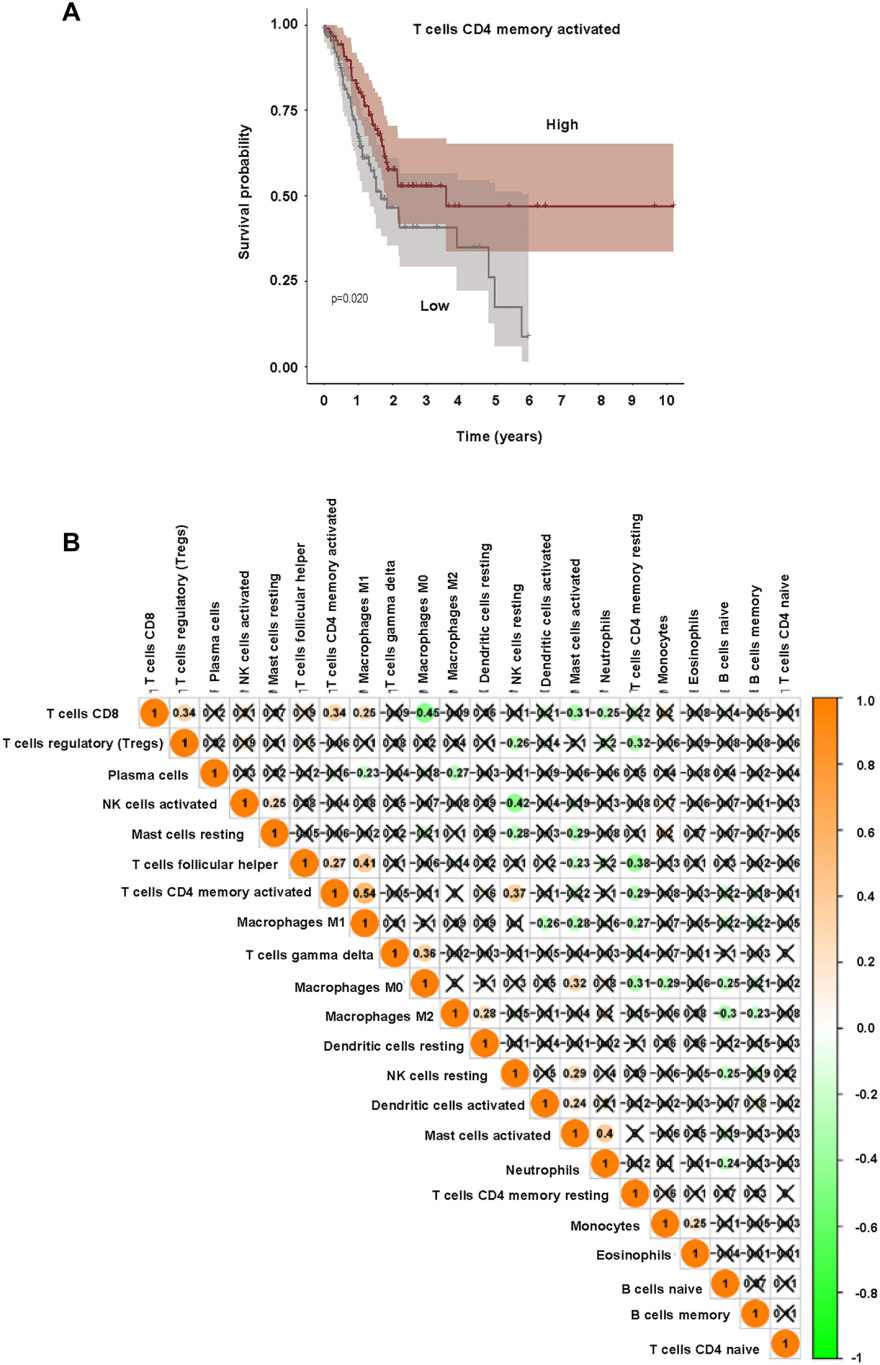
The correlation of immune cells and STAD. (**A**) T cells CD4 memory activated and survival in STAD. (**B**) Co-expression patterns among fractions of immune cells.

**Figure 7:**
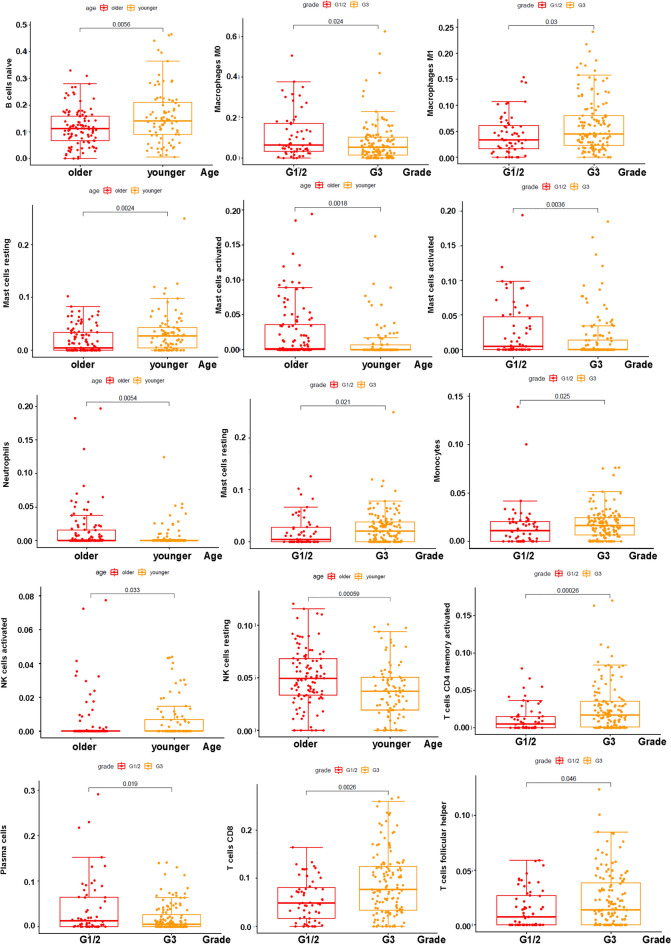
Correlation between immune cells and clinical features.

The immune cell content in the samples was shown in Fig. 5A. It was depicted that B cells naïve, T cells CD4 memory resting, Macrophages M0, M1, M2 were higher in tumor cells, while B cells memory, plasma cells, T cells CD8, T cells CD4 memory activated and Monocytes were lower in tumor cells (Fig. 5B). The heatmap was also demonstrated that various immune cells were differently expressed in tumor cells (Fig. 5C). CD4 memory activated T cells was associated with survival probability. High number of CD4 memory activated T cells had a good survival probability (Fig. 6A). The association among 22 different types of immune cells was presented (Fig. 6B). The expression of B cells naïve, Mast cells resting and NK cells activated was higher in younger than in older age, while the expression of Mast cells activated, Neutrophils, NK cells resting was lower than in older age (Fig. 7). The expression of Macrophage M0, Mast cells activated, and Plasma cells was higher in G1/2 than in G3 grade, while the expression of Macrophage M1, Mast cells resting, Monocytes, T cells CD4 memory activated, T cells CD8, and T cells follicular helper was lower in G1/2 than in G3 grade (Fig. 7).

### Monocytes and neutrophils in gastric cancer

**Figure 8:**
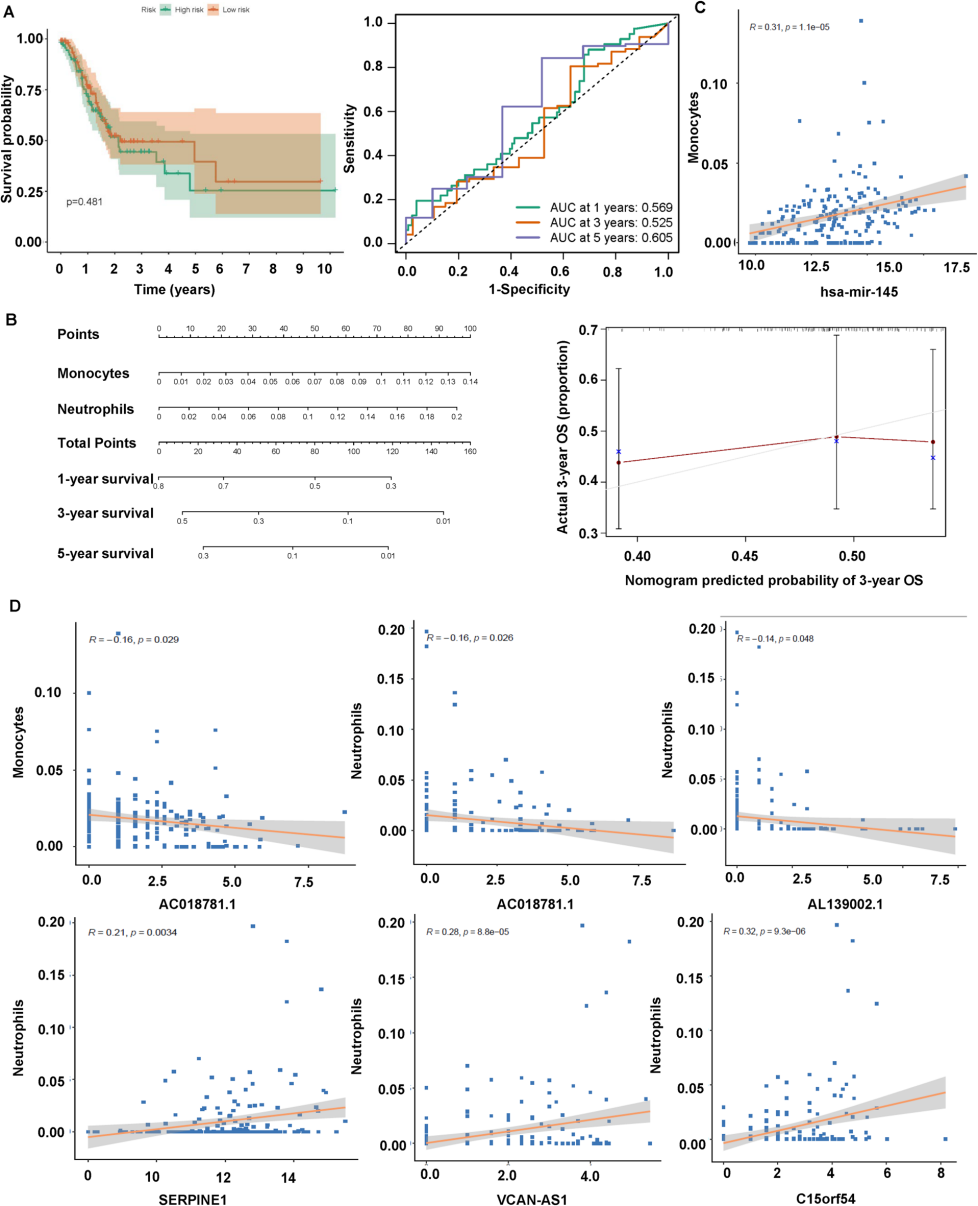
The association of immune cells and survival in STAD. (**A**) Survival probability of high and low-risk groups (left). AUC curve at 1, 3, 5 years (right). (**B**) Nomogram for predicting patients’ outcome based on immune cells (left). Calibration curves for assessing the discrimination and accuracy of the nomogram (right). (**C–D**) The association between immune cells and prognostic genes.

**Figure 9:**
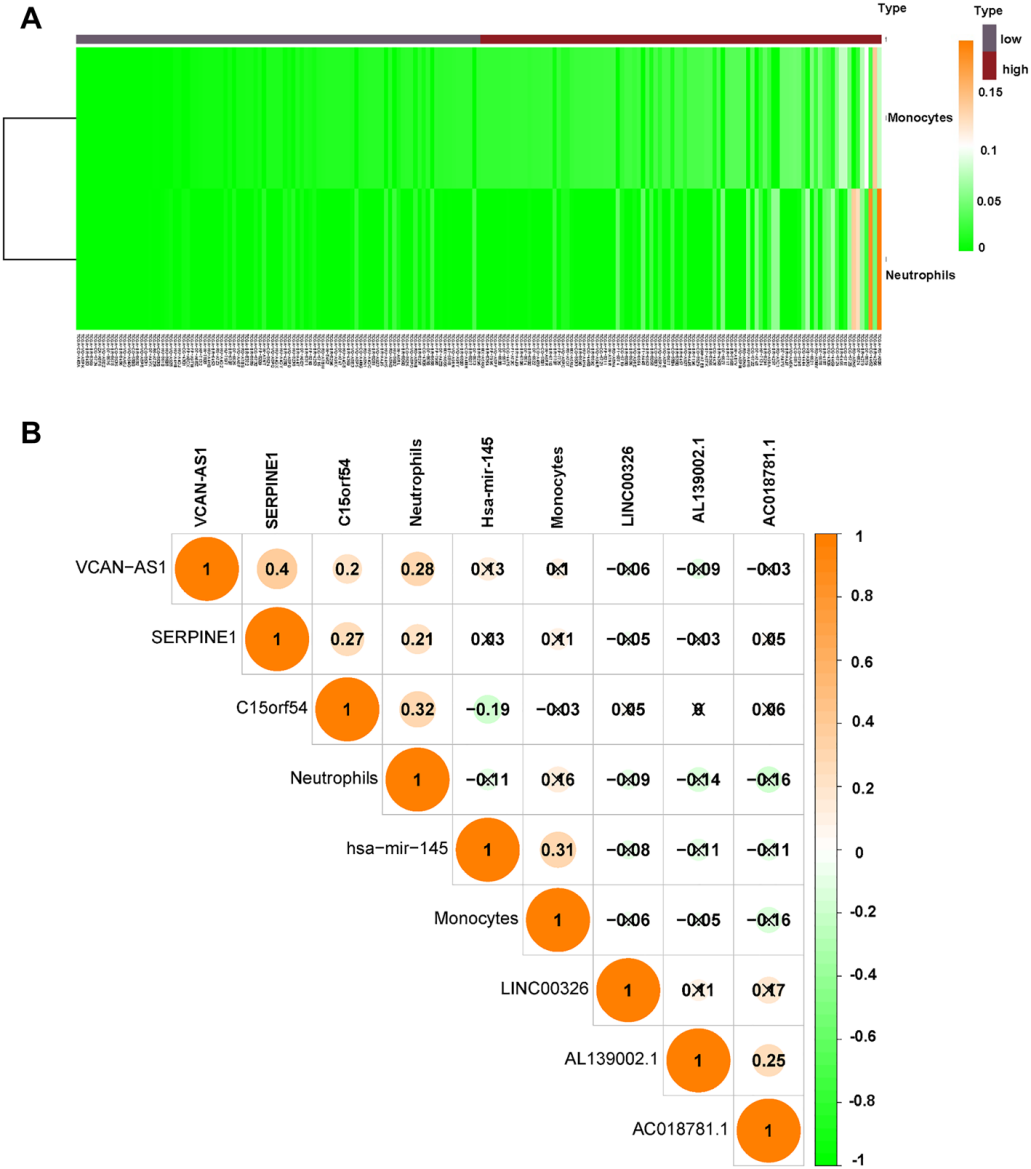
The association of immune cells and survival in STAD. (**A**) Heatmap of the immune cells (monocytes and neutrophils) in high-risk and low-risk groups. (**B**) correlation matrix of immune cells and genes.

Next, we found no significant difference for survival probability between the high-risk group and the low-risk group (Fig. 8A). The data from AUC curve showed that the nomogram survival prediction model had a reasonable accuracy. The AUC values at 1 year, 3 years, and 5 years were 0.569, 0.525, and 0.605, respectively (Fig. 8A). Cox regression analysis obtained two predictable related immune cells, Monocytes and Neutrophils. The Nomogram was established based on the immune cells, which predicts survival rates for the first, 3 to 5 years. The immune cells in this nomogram model were Monocytes and Neutrophils (Fig. 8B). Moreover, the calibration curve for nomogram models demonstrated a consistent agreement between the actual survival rate and the predicted 3 years overall survival rate, implying that this model was accurate in its predictions (Fig. 8B). In addition, hsa-miR-145 and AC018781.1 were associated with Monocytes, while AC018781.1, AL139002.1, SERPINE1, VCAV-AS1 and C15orf54 were associated with Neutrophils (Fig. 8C-D). In the high-risk group, Monocytes and neutrophils were highly expressed (Fig. 9A). Pearson’s correlation assessed the association between biomarkers and immune cells. C15orf54 had the strongest relationship with Neutrophils with a coefficient of 0.32. VCAN-AS1 had a good association with Neutrophils with a coefficient of 0.28. In addition, hsa-miR-145 had a stronger relationship with Monocytes with a coefficient of 0.31 (Fig. 9B).

## Discussion

Although the incidence and mortality of gastric cancer have decreased in recent years, gastric cancer is still one of the most common malignant tumors in the world and causes a heavy medical burden globally^3^. To identify potential biomarkers associated with the diagnosis, treatment and prognosis of gastric cancer, comprehensive bioinformatics analysis may help to achieve this goal and develop personalized treatment for gastric cancer patients. Based on RNA expression profiles from TCGA, 980 lncRNAs, 104 miRNAs and 1639 mRNAs were identified, which were differentially expressed between tumors and normal tissues. The current work used lncRNA-associated ceRNA to identify key biomarkers that were related to the prognosis of gastric cancer. The lncRNAs VCAN-AS1, AL139002.1, LINC00326, AC018781.1, and C15orf54 served as hub nodes in the ceRNA network, which targeted other miRNAs and mRNAs.

LncRNAs have also been considered as an underappreciated novel therapeutic target for gastric cancer^43^. Their diverse roles in cancer progression offer new opportunities to disrupt metastasis in clinical settings^44^. LncRNAs play important roles in gene regulation, including regulation of gene activation and silencing, X chromosome inactivation, alternative splicing, and post-translational regulation. mRNAs, miRNAs, and lncRNAs are linked in an intricate network of crosstalk by competing endogenous RNAs^45^. LncRNA VCAN-AS1 has been reported to promote the malignant tumor behaviors via modulating the miR-106a-5p-involved STAT3/HIF-1α axis in breast cancer^46^. Two studies also validated that lncRNA VCAN-AS1 was abnormally expressed in gastric cancer by constructing the ceRNA regulatory network^47,48^. Feng et al.^49^ reported that lncRNA VCAN-AS1 contributed to gastric cancer progression via regulation of p53 expression. Hence, lncRNA VCAN-AS1 might be a potential factor to influence gastric cancer development and progression. Due to the unclear role of lncRNA VCAN-AS1 in TIME, further investigation is required to determine the functions of VCAN-AS1 in regulating TIME in gastric cancer.

One study has confirmed that AL139002.1 sponged miR-490-3p and regulated the expression of Hepatitis A virus cellular receptor 1 (HAVCR1) in gastric cancer, contributing to the development of gastric cancer^50^. In addition, LINC00326 has been found to regulate hepatocarcinogenic lipid metabolism^51^. In non-small cell lung carcinoma, overexpression of LINC00326 attenuated tumor progression via blockade of Wnt/β-catenin pathway through miR-657/DKK2 axis^52^. Liu et al.^53^ used a competing endogenous RNA network and found that 3 lincRNAs, C15orf54, ADAMTS9-AS1 and AL391152.1, were involved in survival rate of gastric cancer patients. LncRNA AC018781.1 was observed to be an independent risk factors for gastric cancer by a bioinformatic and integrated analysis^48^.

In this study, we found the important role of miR-145 in gastric cancer. In line with this finding, numerous studies have validated the functions of miR-145 in gastric cancer progression^54^. For example, miR-145 inhibited cell proliferation, invasion and migration as well as cell cycle progression via suppression of transcription factor Sp1 in gastric cancer^55^. Xing et al. reported that miR-145 targeted the expression of catenin-δ1, MYO6 and inhibited cell invasion in gastric cancer^56,57^. Chang et al.^58^ found that miR-145 induced the antiproliferative effects via targeting E2F3 in gastric cancer. Low expression of miR-145-5p was associated with poor prognosis in gastric cancer^59^. It is clear that the role of miR-145 has been well studied in gastric cancer.

SERPINE1 gene has been studied in a variety of human cancers, including gastric cancer. One group reported a SERPINE1-based immune gene signature, which can predict immunotherapy response and patient prognosis in gastric cancer^60^. Another group uncovered that SERPINE1 enhanced malignant progression and associated with poor prognosis in gastric cancer patients^61^. Several analyses support SERPINE1 as a potential prognostic biomarker in gastric cancer^62–64^. One study used data mining in combination with bioinformatics dissected that SERPINE1 was associated with immunoinfiltration and might be a diagnosis and prognosis biomarker in stomach adenocarcinoma^65^. High SERPINE1 expression group presented higher immune cells’ expression, including CD4+ T cells, neutrophils, CD8+ T cells, macrophages and B cells. Moreover, SERPINE1 was associated with immune cells in the TIME of stomach adenocarcinoma^65^. Another study discovered that SERPINE1 expression was correlated with several types of immune cells, such as neutrophils, macrophages, CD4+ T cells, CD8+ T cells, B cells, and dendritic cells in clear cell renal cell carcinoma^66^.

Recently, evidence suggested that SERPINE1 was associated with immune infiltrates in gastric cancer^67^. SERPINE1 enhanced the inhibitory TIME, which was positively associated with the infiltration level of neutrophils, macrophage M2, resting NK cells, and activated mast cells. SERPINE1 was negatively associated with plasma cells and B cell memory in gastric cancer^67^. Consistently, in our study, we identified that SERPINE1 expression was positively correlated with neutrophils in gastric cancer.

CD4+ regulatory T cells were identified to be associated with survival in gastric cancer^68^. In gastric cancer patients, CD4+ T cells and regulatory T cells were enriched, and lower expression of miR-128-3p was correlated with overall survival. Moreover, miR-128-3p targeted the expression of IL16 in gastric cancer^68^. One group reported that high infiltration of CD4+ T cells was linked to worse overall survival in gastric cancer^69^. Another group revealed that CD4+ memory T cell-related genes were correlated with clinical overall survival in patients with gastric cancer^70^. Additionally, lncRNAs, including A2M-AS1, C2orf27A, and ZNF667-AS1, impaired activation of CD4+ T cells and affected prognosis of gastric cancer patients^71^. In gastric cancer patients with FLOT chemotherapy (5-Fluorouracil, Leucovorin, Oxaliplatin and Docetaxel), CD4+/CD8+ lymphocyte ratio was elevated and predicted favourable therapy response^72^. In our study, CD4 memory activated T cells were linked to survival of gastric cancer patients. Without a doubt, more exploration is necessary to determine the mechanism of CD4 T cells-mediated survival in gastric cancer.

In summary, we successfully identified lncRNAs closely related to the occurrence and development of gastric cancer based on the TCGA database. Based on these lncRNAs, a ceRNA network based on the lncRNA–miRNA–mRNA regulatory mechanism was constructed. Several lncRNAs, miRNAs and mRNAs associated with gastric cancer prognosis were screened in the ceRNA network by survival analysis. These indicators provide new targets for the prognosis evaluation of gastric cancer patients. At the same time, our study also confirmed the relationship between immunity and lncRNA-based ceRNA regulatory network. This study will enable us to identify more useful targets to develop effective treatment strategies for gastric cancer. It has several limitations in this study. In TCGA database, there are lower numbers of control groups compared with the tumor groups. The discrepancy between the control group and the experimental group could lead to potential bias in the analysis results. In addition, it is important to mention that in vitro and in vivo experiments are necessary to validate our findings in the future.

## Materials and methods

### Data colleting from TCGA

RNA-sequencing data, miRNA profiling and clinical information of STAD were downloaded from the TCGA database (https://portal.gdc.cancer.gov/). Illumina HiSeqRNASeq platforms were used to obtain mRNA and lncRNA data. HiSeqmiRNASeq platforms were used to collect miRNA data. Data were collected for gastric cancer samples and normal samples. Data were further organized by ID conversion, filtering, merging, correction and clinical information. Demography, histology, tumor stage and TNM of the STAD were obtained (Supplementary Table 1 and supplementary file 1). Pre-filtered low count genes (rowMeans(data) > 1) were used to preprocess RNA sequencing profiles. We converted the downloaded data into count format through R, so as to analyze these samples and screen out differentially expressed RNAs. EdgeR (v.3.28.0) is an R package dedicated to analyzing DEGs (differentially expressed genes). Standard settings for DEGs were FDR < 0.01 and log fold change (FC) > 2. DElncRNA (differentially expressed lncRNA), DEmRNA (differentially expressed mRNA) and DemiRNA (differentially expressed miRNA) were visualized using the ggplot package. The Ensembl database was used to annotate genes in RNAseq, and we excluded RNAs not included in the Ensembl database. Afterwards, we extracted lncRNA expression profiles and mRNA expression profiles from the RNAseq expression matrix.

### Construction of CeRNA regulatory network

To further understand the role of DERNA (differentially expressed RNA) in gastric cancer, we constructed a ceRNA regulatory network between lncRNA-miRNA-mRNA to explore the relationship between them. Then, a ceRNA network was built using the hypergeometric test and correlation analysis to identify the differentially expressed miRNAs that can control lncRNAs and mRNAs (*p* < 0.05 as the filter threshold criteria). DElncRNAs, DEmiRNAs and DEmRNAs were incorporated into ceRNA regulatory network. We visualized the ceRNA regulatory network with Cytoscape. PPI network of mRNAs involved in the ceRNA network were generated by STRING^73^.

### GO analysis and KEGG pathway analysis

Functional analysis of genes from different organisms, which includes three gene ontology of cellular component (CC), molecular function (MF) and biological process (BP), was further conducted through the Gene ontology (GO) database^74^. The clusterProfiler (v.3.14.3) package was adopted to perform GO functional annotation and KEGG pathway analysis of mRNAs in the ceRNA network^75^.

### Prognosis analysis of related genes

The clinical information of gastric cancer patients was downloaded from TCGA database and then we extracted the survival information of patients (survival time and survival status of patients)^76^. Subsequently, survival information with the expression matrix of RNAs in the ceRNA network was combined. To determine whether there is a relationship between biomarker expression and STAD survival, Kaplan-Meier (K-M) survival analysis was performed^77^. The next step was to find significant factors in the original Cox model that had prognostic significance and to incorporate those into the reduced Cox proportional hazard model^78^. In order to test the accuracy of the created multifactorial model, LASSO regression uses contraction to lower the data value to a particular point^79^. Ultimately, a nomogram was developed using the multivariate model to forecast the prognosis of STAD. The nomogram might make it possible for doctors to assign each biomarker a prognosis value based on how it expresses^80^. Indicators of prognosis and total 3- and 5 years survival may be found in the sum of the individual values. The accuracy of the nomograms was evaluated using receiver operating characteristic curve (ROC) analysis and calibration^81^.

### Immune infiltration in STAD

Gene expression data were used by CIBERSORT to estimate the proportion and abundance of different types of immune cells in tumor and normal groups^82^. To assess the percentages of 22 immune cell types in STAD samples, we used CIBERSORT in this instance. Only instances with CIBERSORT results of *P* ≤ 0.05 were considered for further analysis. Important immune cells in tumor samples were distinguished from non-malignant samples using Wilcoxon rank-sum^83^. Next, K-M survival analysis was used to see whether the number of particular immune cells was related to overall STAD survival^84^. Different immune cells were incorporated into a Cox proportional hazard model following LASSO regression analysis, and a nomogram for STAD prognosis prediction was created^85^. The bias and precision of the nomogram were evaluated using the concordance index of the Cox mode.

### Association between selected RNAs and immune cells

To examine the immune cells that are connected to survival, univariate Cox regression and Kaplan-Meier survival analysis were employed^77^. In parallel, multivariate Cox regression analysis and Lasso regression were used to create the final immune cell model^86,87^. An equation that forecasts medical outcomes was graphically represented as a nomogram. With nomograms, patients accumulate points based on the severity of their risk factors^85^. The fold difference in gene expression between tumor tissues and normal tissues served as the basis for the risk factors of two nomograms that we constructed for the study. Then, we forecast the patients’ 1-, 3-, or 5 years survival rate.

### Supplementary Information

Supplementary Information.

Supplementary Table 1.

## Data Availability

The data of this study are available from the corresponding author upon reasonable request.

## Abbreviations

ceRNA: Competing endogenous RNA
DElncRNA: Differentially expressed lncRNA
DEmRNA: Differentially expressed mRNA
DemiRNA: Differentially expressed miRNA
DERNA: Differentially expressed RNA
FENDRR: FOXF1 adjacent noncoding developmental regulatory RNA
FLOT: 5-Fluorouracil, leucovorin, oxaliplatin and docetaxel
HAVCR1: Hepatitis A virus cellular receptor 1
GO: Gene ontology
KEGG: The kyoto encyclopedia of genes and genomes
lncRNA: Long noncoding RNA
miRNA: Microrna
mRNA: Messenger RNA
NKILA: Nuclear factor-κB-interacting lncRNA
OSCC: Oral squamous cell carcinoma
ROC: Receiver operating characteristic
SERPINE1: Serine protease inhibitor clade E member 1
TIME: Tumor immune microenvironment
TCGA: The cancer genome atlas
VCAN-AS1: VCAN antisense RNA 2
