# Supplementary material for: Integrated bioinformatics analysis of noncoding RNAs with tumor immune microenvironment in gastric cancer
Source: Sci Rep. 2023 Sep 11;13:15006. doi: 10.1038/s41598-023-41444-3 (PMC10495442; doi:10.1038/s41598-023-41444-3)
Supplement: Supplementary file 2 — Supplementary Table 1. [file 41598_2023_41444_MOESM2_ESM.docx]

| Age | number | Gender | number | Grade | Number. | Stage | number | T | number | N | number | M | number |
| --- | --- | --- | --- | --- | --- | --- | --- | --- | --- | --- | --- | --- | --- |
| >60 | 296 | Male | 285 | G1 | 12 | 1 | 2 | T1 | 2 | N0 | 132 | M0 | 391 |
| ≤60 | 142 | Female | 158 | G2 | 159 | 1a | 16 | T1a | 16 | N1 | 119 | M1 | 30 |
| Unknow | 5 |  |  | G3 | 263 | 1b | 41 | T1b | 41 | N2 | 85 | MX | 22 |
|  |  |  |  | GX | 9 | 2 | 33 | T2 | 33 | N3 | 32 |  |  |
|  |  |  |  |  |  | 2a | 41 | T2a | 41 | N3a | 49 |  |  |
|  |  |  |  |  |  | 2b | 56 | T2b | 56 | N3b | 7 |  |  |
|  |  |  |  |  |  | 3 | 3 | T3 | 3 | Nx | 17 |  |  |
|  |  |  |  |  |  | 3a | 79 | T3a | 79 | unknow | 2 |  |  |
|  |  |  |  |  |  | 3b | 63 | T3b | 63 |  |  |  |  |
|  |  |  |  |  |  | 3c | 38 | T3c | 38 |  |  |  |  |
|  |  |  |  |  |  | 4 | 44 | T4 | 44 |  |  |  |  |
|  |  |  |  |  |  | unknow | 27 | unknow | 27 |  |  |  |  |

Table 1: The clinical characteristics of 443 gastric cancer patients.
